# A randomized, double blinded, placebo controlled trial of oral dydrogesterone supplementation in the management of preterm labor

**DOI:** 10.1038/srep20638

**Published:** 2016-02-09

**Authors:** Wilasinee Areeruk, Vorapong Phupong

**Affiliations:** 1 Department of Obstetrics and Gynecology, Faculty of Medicine, Chulalongkorn University, Rama IV Road, Pathumwan, Bangkok 10330, Thailand

## Abstract

The primary aim of this study was to evaluate the effect of oral dydrogesterone on the recurrent uterine contraction in preterm labor. The secondary aims were to evaluate latency period, gestational age at delivery, pregnancy outcomes, neonatal outcomes, compliance and side effects. A randomized, double blinded, placebo controlled trial was conducted. Forty-eight pregnant women at 24–34 weeks gestation with preterm labor were either randomized to study group receiving tocolytic treatment combined with oral dydrogesterone (20 mg daily) or to placebo group receiving tocolytic treatment combined with oral placebo. Recurrent rates of uterine contraction were comparable between groups (87.5% vs 91.7%, p = 0.64). Latency periods were not different between dydrogesterone and placebo group (32.7 ± 20.2 days vs 38.2 ± 24.2 days, p = 0.39). There were also no differences in gestational age at delivery, pregnancy outcomes, neonatal outcomes, compliance and side effects. Adjuvant treatment with oral dydrogesterone 20 mg/day could not decrease the rates of recurrent uterine contraction and prolong latency period in preterm labor management when compared to placebo.

Preterm labor, defined as spontaneous labor occurring before 37 weeks gestation, is one common obstetric complication that often leads to preterm birth. Preterm labor precedes 40–50% of preterm births which is defined as birth between 20 weeks of gestation and before 37 weeks of gestation^1^. Preterm birth is associated with neonatal morbidities such as respiratory distress syndrome (RDS), intraventricular hemorrhage (IVH), necrotizing enterocolitis (NEC), neonatal sepsis, cerebral palsy and neonatal mortality. Preterm births account for 70% of neonatal deaths and 36% of infant deaths as well as 25–50% of cases of long-term neurological impairment in children^1^. There has been an increase in the incidence of preterm birth over the past decade^2^. Management of preterm labor consists of tocolysis and corticosteroids. Tocolysis is used for 48 hours for treatment of preterm labor to allow adequate time for corticosteroid administration to improve fetal lung maturity^3^.

Several trials have been conducted to focus on the prevention of preterm labor. One of the most promising agents to aid in prevention of preterm labor is progesterone. It has been investigated for its preventative role in preterm labor and has been included in recent guidelines that recommend progesterone as a primary prevention agent in singleton pregnancy with a prior spontaneous preterm birth or short cervical length^4^.

Although supporting evidence is still unclear for the prevention of preterm birth in those whose cervix has dilated, symptoms of preterm labor, cerclage and uterine anomaly, progestogen has been used extensively over the past decade^5^. There have been many trials investigating the use of progestogen for maintaining pregnancy in preterm labor patients. Some studies found prolongation of the latency period^6^,^7^,^8^,^9^,^10^. while one study did not^11^. Most trials used vaginal progesterone despite its difficulty to administer and bothersome side effects.

Dydrogesterone, a stereoisomer of progesterone, appears to be a highly selective progestin which, due to its retrostructure, binds almost exclusively to the progesterone receptor. Dydrogesterone has a good safety and tolerability profile. It is structurally and pharmacologically similar to natural progesterone and has good oral bioavailability with few side effects. After oral administration, it is generally absorbed reaching a maximum serum concentration within 2–5 hours, and displays stable plasma level^12^. The dosage of 20 mg/d is an equivalent dose to 200 mg vaginal progesterone^12^.

To date, there has been no study to investigate the use of dydrogestogen for maintaining pregnancy in preterm labor. Thus, we focused mainly on secondary prevention of preterm labor. The primary aim of this study was to evaluate the effect of dydrogesterone on the recurrent uterine contraction in preterm labor. Secondary aims were to evaluate latency period, pregnancy outcomes, neonatal outcomes, compliance and side effects.

## Subjects and Methods

This study was a randomized, double blinded, placebo controlled trial conducted at the Department of Obstetrics and Gynecology, Faculty of Medicine, Chulalongkorn University, Bangkok, Thailand, between August 1, 2014 and June 30, 2015. This study was approved by the Research Ethics Committee of the Faculty of Medicine, Chulalongkorn University. The methods were performed in accordance with approved guidelines. Written informed consent was obtained from all participants. This clinical trial was registered at ClinicalTrials.gov (Clinical trials registration: NCT02262481 date October 3, 2014).

Pregnant women aged 18 to 45 years with singleton pregnancy presented to our hospital with the diagnosis of preterm labor between 24 to 34 weeks of gestation with intact membranes were invited to join this study. Preterm labor was defined as having regular uterine contraction accompanied by changes in cervical dilatation or effacement^1^. Pregnant women with fetal or maternal conditions that required immediate delivery (such as fetal distress, chorioamnionitis, placenta previa, abruptio placenta and severe preeclampsia), cervical dilatation ≥5 cm., fetal anomalies, contraindications to tocolytic drugs and known allergies to progestogen were excluded.

After the study was approved, eligible women who gave informed consent were enrolled. Uterine contractions were measured using a tocometer. Cervical dilatation and effacement were assessed by pelvic examination. Uterine contraction and cervical effacement and dilatation were recorded. Baseline investigations including complete blood count, urinary analysis, cervical swab culture as well as ultrasonography to evaluate the estimated fetal weight were performed. All women received dexamethasone 6 mg intramuscularly every 12 hours for a total of 4 doses and received 48 hours of tocolytic oral nifedipine. Dosage of nifedipine was 10–20 mg every 6 hours.

Pregnant women were randomized into two groups: treatment or placebo group. A randomization scheme was generated by random number table using a block-of-four technique. The co-investigator, who had no contact with patients, generated the allocation sequence prior to the study. Nurses enrolled and assigned participants to their respective groups. Drug and placebo were prepared prior to the study by a pharmacist who had no involvement in the study. Each dydrogesterone 10 mg tablet was put into a capsule and no drug was placed in a placebo capsule. As soon as a study subject met the inclusion criteria, nurses proceed to select a sequentially numbered opaque envelope.

Opaque envelopes containing 28 or 14 capsules of dydrogesterone or placebo (identical in size, shape and color) were sequentially labeled. A 28-capsule opaque envelope was used for participants with 2-week follow-up and a 14-capsule opaque envelope was used for participants with 1-week follow-up. To ensure randomization, each envelope was distributed in sequential numerical order. Both health care providers and study participants were masked to treatment assignment. Dydrogesterone (Duphaston^®^, 10 mg per tablet) (Abbott, Bangkok, Thailand) was assigned to the treatment group and corresponding placebo to the placebo group. Drug was administered at the same time (from the beginning) of oral nifedipine administration. Drug dose was one tablet, two times a day after meal. Treatment was continued until delivery or 37 weeks of gestation. Treatment assignment was not revealed until data collection was completed. Participant was admitted in the labor room until uterine contraction stopped. Participant recorded uterine contraction in a diary chart and had 2-week interval follow-ups until delivery. If gestational age reached 36 weeks or more, participant had a 1-week interval follow-up until delivery. Participants were asked to return the envelopes and diary chart at each follow-up and at the end of study to evaluate compliance. Good compliance was defined as participant took all medication.

The primary outcome was to assess the recurrent uterine contractions after completing a 48-hour course of tocolysis. Secondary outcomes were to assess latency period (the time since onset of preterm labor until delivery), gestational age at delivery, pregnancy outcomes, neonatal outcomes, compliance and side effects. Neonatal outcomes included birth weight, low birth weight, Apgar scores, neonatal intensive care unit (NICU) admission, days of neonatal hospitalization, birth asphyxia, respiratory distress syndrome (RDS), intraventricular hemorrhage (IVH), necrotizing enterocolitis (NEC), neonatal sepsis and neonatal death. The recurrent uterine contraction was defined as regular uterine contraction occurred at least 4 times in 20 minutes for at least 1-hour duration after stopping tocolysis and cervix was dilated at least 1 cm.

Sample size calculation was based upon the recurrent uterine contractions and the latency period. The rate of recurrent uterine contractions from pilot study was 80%. We expected a 50% decrease rate of recurrent uterine contraction. With adjustments for a withdrawal rate of 10%, a minimum of 24 women in each group were required to detect statistical difference (α = 0.05, β = 0.2). We also expected a 14-day latency period. From a previous study, the latency period was 21.2 ± 16.3 days in the control group^10^. With adjustments for a withdrawal rate of 10%, a minimum of 24 women in each group were required to detect statistical difference (α = 0.05, β = 0.2). Thus, total 48 women were required in this study.

### Statistical analysis

SPSS version 22 (SPSS Inc, Chicago, IL, USA) was used for statistical analysis. Chi-square test and Fisher-exact test for categorical variables, independent *t*-test for continuous variables, and Mann-Whitney U test for nonparametric variables were used when appropriate. A p < 0.05 was considered statistically significant. Analysis of the trial was conducted in intent-to-treat (ITT) analysis.

## Results

**Figure 1:**
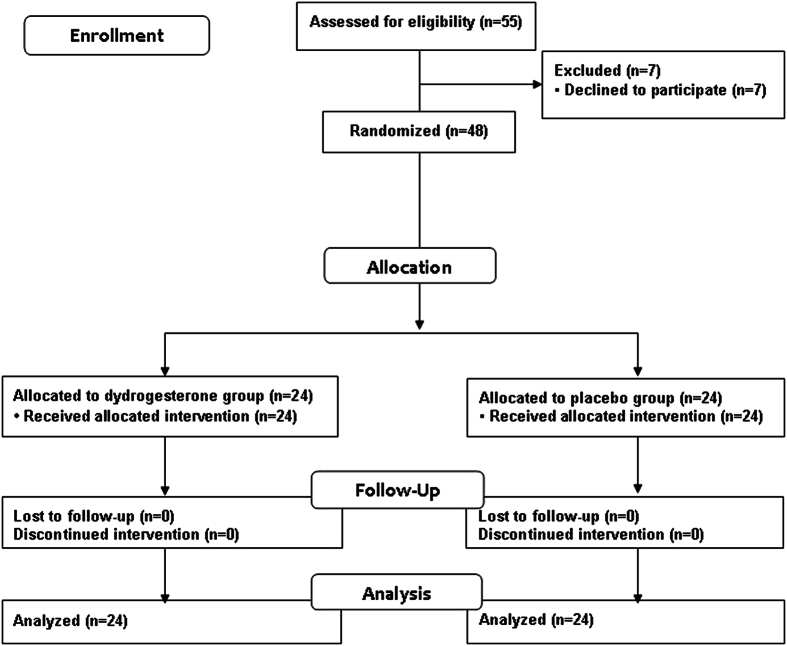
Profile of patient follow-up following randomization to either dydrogesterone or placebo group.

Fifty-five women were enrolled, 7 women refused to participate in the study. The remaining 48 women were randomly assigned to two groups: 24 received dydrogesterone (20 mg per day) and 24 received placebo (Fig. 1). None of the women were lost to follow-up. For demographic characteristics, there were no significant differences between the groups in respect to age, gravidity, parity, gestational age, pre-pregnancy body mass index (BMI) and history of preterm birth (Table 1).

Rates of recurrent uterine contraction were comparable between groups (87.5% vs 91.7%, p = 0.64). Latency periods were not different between dydrogesterone and placebo group (32.7 ± 20.2 days vs 38.2 ± 24.2 days, p = 0.39) (Table 2). Gestational age at delivery, percentage of delivery before 34 weeks, percentage of delivery before 37 weeks and mode of delivery did not differ between the groups (Table 2).

Table 3 demonstrates compliance and side effects. Compliance was good with no different between the two groups. There were also no differences in side effects between groups. Nausea and vomiting occurred in only one case in the dydrogesterone group.

Neonatal outcomes are shown in Table 4. Neonatal birth weight, low birth weight and apgar scores <7 at 1 and 5 min did not differ between the groups. Additionally, differences between groups in respect to RDS, IVH, NEC, sepsis, apnea of prematurity, transient tachypnea of new born (TTNB), NICU admission and days of neonatal hospitalization were not significant. There was no neonatal mortality in this study.

## Discussion

This randomized, double blinded, placebo controlled trial evaluated the efficacy of adjuvant oral dydrogesterone in patients treated with nifedipine tocolysis for preterm labor. This study shows that the recurrent uterine contraction rates and the latency period were not different between the dydrogesterone and placebo groups. Similar results were found in terms of gestational age at delivery, pregnancy outcomes, neonatal outcomes, compliance and side effects.

In the present study, the latency period was not different between the dydrogesterone and placebo groups (32.7 days vs 38.2 days, p = 0.39). This finding was similar to the study from Noblot *et al*. They evaluated the use of maintenance tocolysis with oral micronized progesterone, but the results showed no significant difference in the latency period between progesterone and placebo groups (6.0 vs 6.4 days)^11^.

Results of this present study differed from previous studies that evaluated vaginal micronized progestogen on threatened preterm labor^6^,^7^,^8^,^9^,^10^. Sharami *et al*. demonstrated that prophylactic administration of 200 mg vaginal progesterone after successful tocolysis by intravenous magnesium sulfate in patients with preterm labor could prolong the pregnancy period. Mean latency periods were significantly longer in the progesterone group (23.88 days) compared with the placebo group (16.67 days, p = 0.004)^6^. Ariea *et al*. used 200 mg vaginal progesterone starting on the day after terminating atosiban as a tocolysis. They found that patients receiving progesterone had a longer latency period (55 vs 38 days, p = 0.02)^7^. Borna and Sahabi found that the use of 400 mg vaginal progesterone suppository after successful parenteral magnesium sulfate tocolysis was associated with longer latency when compared to no medication (36.1 vs 24.5 days, p = 0.03)^8^. Bomba-Opon *et al*. retrospectively reviewed the use of 200 mg vaginal progesterone after tocolysis (fenoterol and verapamil) in threatened preterm labor and found that it is associated with prolongation of pregnancy when compared to no medication (7.6 versus 6.3 weeks, p = 0.039)^9^. Arikan *et al*. found that the treatment of threatened preterm birth with tocolytics (ritodrine) combined with intravaginal 200 mg micronized natural progesterone significantly prolonged pregnancy when compared to no medication (32.1 vs 21.2 days, p < 0.05)^10^. The difference of results between studies may be due to the difference of drug administration route and kind of drug.

The mechanisms of progesterone in prolonging pregnancy are not clearly known, but a previous study found that progesterone withdrawal directly precedes progression of phase 1 into phase 2 parturition. Progesterone maintains uterine quiescence by various mechanisms including relaxation of myometrial smooth muscle, blocking the action of oxytocin, inhibition of the formation of gap junctions, decreasing the concentration of myometrial oxytocin receptors and inhibiting prostaglandins production by amnion-chorion-decidua^8^. However, there has been no literature on the effect of dydrogesterone on uterine contractions. Thus, dydrogesterone does not have a role as tocolytic agent. In the present study, recurrent uterine contraction after completing a 48-hour course of tocolysis was lesser in the dydrogesterone group than in the placebo group (87.5% vs 91.7%), but there was no statistical significance.

There was also no difference between the two groups in gestational age at delivery, pregnancy outcomes and neonatal outcomes in the present study. This might explain that the addition of oral dydrogesterone did not increase the clinical benefits of tocolysis (nifedipine).

The strength of this study was that it is the first randomized, double blinded, placebo controlled trial conducted to evaluate the efficacy of oral dydrogesterone for both tocolytic and maintenance treatment of preterm labor. Dydrogesterone was chosen for investigation in order to avoid the disadvantage of oral progesterone which has a wide variation of absorption and bioavailability among individuals, even in micronized form. The dosage of 20 mg/day was selected because it was an equivalent dose to 200 mg vaginal progesterone from Ariea *et al*.’s study^7^. The limitation of this study was that it is underpowered to evaluate the secondary outcomes due to a small sample size. A future randomized controlled trial with larger sample size and a higher dose of dydrogesterone should be conducted to better evaluate the benefits of oral dydrogesterone as a secondary prevention of preterm labor.

In conclusion, the adjuvant treatment with oral dydrogesterone 20 mg/day could not decrease the rates of recurrent uterine contraction and prolong latency period in preterm labor management when compared to placebo. There were no differences in gestational age at delivery, pregnancy outcomes, neonatal outcomes compliance and side effects.

## Additional Information

**How to cite this article**: Areeruk, W. and Phupong, V. A randomized, double blinded, placebo controlled trial of oral dydrogesterone supplementation in the management of preterm labor. *Sci. Rep.* **6**, 20638; doi: 10.1038/srep20638 (2016).

## Figures and Tables

**Table 1:** Demographic characteristics.

Characteristics	Dydrogesterone group(n = 24)	Placebo group (n = 24)	P value
Age	31.0 ± 6.3	27.7 ± 6.9	0.09
Gravidity			0.37
Primigravida	7 (29.2%)	10 (41.7%)	
Multigravida	17 (71.8%)	14 (58.3%)	
Parity			0.56
Nulliparous	9 (37.5%)	11 (45.8%)	
Multiparous	15 (62.5%)	13 (54.2%)	
History of preterm birth	3 (12.5%)	2 (8.3%)	1.00
BMI (kg/m^2^)	21.4 ± 2.4	21.5 ± 3.4	0.90
GA at admission (weeks)	32.3 ± 1.6	31.4 ± 2.4	0.16
Median of cervical dilatation (cm)	1	1	0.92

Data presented as mean ± SD or n (%).

BMI: body mass index, GA: gestational age.

**Table 2:** Recurrent of uterine contraction, latency periods, gestational age at delivery, mode of delivery and pregnancy outcomes.

	Dydrogesterone group (n = 24)	Placebo group (n = 24)	P value
Recurrent of uterine contraction	21 (87.5%)	22 (91.7%)	0.64
Latency periods (days)	32.7 ± 20.2	38.2 ± 24.2	0.39
GA at delivery (weeks)	36.9 ± 2.6	36.9 ± 2.3	0.97
Delivery			
<34 weeks	4 (16.7%)	3 (12.5%)	1.00
<37 weeks	8 (33.3%)	9 (37.5%)	0.76
Mode of delivery			0.75
-Vaginal delivery	13 (54.2%)	12 (50%)	
-Cesarean section	11 (45.8%)	12 (50%)	
Pregnancy complications	1 (4.2%)	2 (8.3%)	1.00

Data presented as mean ± SD or n (%)

GA: gestational age.

**Table 3:** Compliance and side effects of medication.

	Dydrogesterone group (n = 24)	Placebo group (n = 24)	P value
Compliance: good	23 (95.8%)	23 (95.8%)	1.00
Side effects			
Nausea/vomiting	1 (4.2%)	0	1.00

Data presented as n (%).

**Table 4:** Neonatal outcomes.

Character	Dydrogesterone group (n = 24)	Placebo group (n = 24)	P value
Sex			0.56
-Male	12 (50%)	14 (58.3%)	
-Female	12 (50%)	10 (41.7%)	
Birth weight (grams)	2812.7 ± 614.3	2817.2 ± 457.1	0.98
Low birth weight	7 (29.2%)	6 (25%)	0.74
Apgar scores			
At 1 minute < 7	2 (8.3%)	1 (4.2%)	1.00
At 5 minutes < 7	0	0	NA
Median day of hospitalization (days)	5 (3, 6)	5 (3, 7)	0.57
Neonatal complications			
RDS	4 (16.7%)	2 (8.3%)	0.67
IVH	0	0	NA
NEC	0	0	NA
Sepsis	3 (12.5%)	1 (4.2%)	0.61
Apnea of prematurity	2 (8.3%)	2 (8.3%)	1.00
TTNB	1 (4.2%)	1 (4.2%)	1.00
NICU admission	1 (4.2%)	0	1.00
Mortality	0	0	NA

Data presented as mean ± SD, median (interquartile) or n (%).

RDS: respiratory distress syndrome, IVH: intraventricular hemorrhage, NEC: necrotizing enterocolitis TTNB: transient tachypnea of new born, NICU: neonatal intensive care unit.
